# Assesment of Thermodynamic Irreversibility in a Micro-Scale Viscous Dissipative Circular Couette Flow

**DOI:** 10.3390/e20010050

**Published:** 2018-01-11

**Authors:** Pranab Kumar Mondal, Somchai Wonwises

**Affiliations:** 1 Department of Mechanical Engineering, Indian Institute of Technology Guwahati, Guwahati 781039, India; 2 Fluid Mechanics, Thermal Engineering and Multiphase Flow Research Lab (FUTURE), Department of Mechanical Engineering, Faculty of Engineering, King Mongkut’s University of Technology Thonburi, Bangkok 10140, Thailand

**Keywords:** entropy generation, viscous dissipation, micro-annulus, concentric-cylinders, degree of asymmetry, Nusselt number, Brinkman number, second law

## Abstract

We investigate the effect of viscous dissipation on the thermal transport characteristics of heat and its consequence in terms of the entropy-generation rate in a circular Couette flow. We consider the flow of a Newtonian fluid through a narrow annular space between two asymmetrically-heated concentric micro-cylinders where the inner cylinder is rotating at a constant speed. Employing an analytical methodology, we demonstrate the temperature distribution and its consequential effects on the heat-transfer and entropy-generation behaviour in the annulus. We bring out the momentous effect of viscous dissipation on the underlying transport of heat as modulated by the degree of thermal asymmetries. Our results also show that the variation of the Nusselt number exhibits an unbounded swing for some values of the Brinkman number and degrees of asymmetrical wall heating. We explain the appearance of unbounded swing on the variation of the Nusselt number from the energy balance in the flow field as well as from the second law of thermodynamics. We believe that the insights obtained from the present analysis may improve the design of micro-rotating devices/systems.

## 1. Introduction

Thermo-fluidic transport associated with a moving surface has been a topic of great importance to researchers in the last few decades. The thermal transport characteristics of heat adhered to the fluid-flow environment differs from other modes of heat transfer characteristics, essentially because of the rise in fluid temperature, which is primarily attributable to the viscous dissipation effect. Importantly, when heat exchange takes place between a moving boundary and the surrounding fluid, the shear heating induced by the movement of the boundary significantly alters the temperature distribution in the flow field, leading to a drastic change in the thermal transport characteristics of the heat. It needs to be mentioned in this context that the effect of viscous heating invites thermodynamic irreversibility into the system, which, in turn, reduces the exergetic efficiency of the devices/systems following the adverse effect of entropy generation [1,2,3].

Understanding of transport phenomena through micro-scale devices, by exploring the several features of the underlying thermo-fluidic transport, has been critically reviewed by several researchers [4,5,6]. It is important to mention in this context that there are many practical applications where heat transfer normally occurs in the course of fluid motion involving moving boundaries [7,8,9]. Particularly, in micro-maching such as micro-drilling, micro-swirling nozzles, micro-milling and micro-piarcing, the heat transfer rate from the moving boundary to the surrounding fluid is of vital importance, allowing these devices to operate at their best thermodynamic efficiency. However, in applications of these kinds, the thermal energy generated due to viscous dissipation in the flow field is not so trivial as to enable it to be safely neglected; rather, this effect alters the heat transfer rates significantly following a drastic change in temperature in the flow field. Therefore, it is very important to take the effect of viscous dissipation into account, while studying convective heat transfer characteristics over small scales [10,11,12,13,14,15].

It is important to mention here that the effect of viscous dissipation acts as a heat source in the flow field, leading to enhancement in the fluid temperature. In precise terms, this effect invites system irreversibility by altering the heat-transfer characteristics of the system [1,3,16,17,18,19]. There are many studies reported in the literature that have critically discussed the effects of viscous dissipation on the forced convective heat transfer in different geometries considered either as constant heat flux or constant wall temperature thermal boundary conditions [8,9,17,19,20,21]. However, few reported studies have considered the movement of either of the boundaries to describe the quantitative relation among the different physical parameters governing thermo-fluidic transport [7,8,9].

Thermal systems and devices are required to sustain different thermal conditions because of their widespread application in different fields. Taking this into consideration, researchers have also investigated the influence of viscous heating developed by a moving surface on the convective transport of heat in a thermal asymmetrical heating environment [8,22,23]. On the other hand, the rotation-induced effect of shear heating may alter thermo-fluidic transport by changing the temperature distribution in the field and hence the thermodynamic efficiency of the systems/devices. In fact, the rotation of the solid boundary might affect system performance non-trivially in the presence of thermal asymmetries, largely attributed to the rich physical interplay between the induced shear-heating effect and the degree of the asymmetrical wall-heating parameter.

Here, we aim to develop an analytical framework for assessing the heat-transfer characteristics for laminar forced convection through an asymmetrically heated annulus of two infinitely long concentric cylinders, where the inner cylinder is rotating at a constant speed. We show the combined effects of the Brinkman number and the degree of asymmetry on the temperature profile and its ultimate consequence on the heat-transfer and entropy-generation rate in the system. In fact, we bring out the non-trivial interplay of the Brinkman number and the degree of asymmetry on the variation of the Nusselt number, leading to the onset of point of singularity.

## 2. Problem Formulation

### 2.1. Physical Considerations

**Figure 1:**
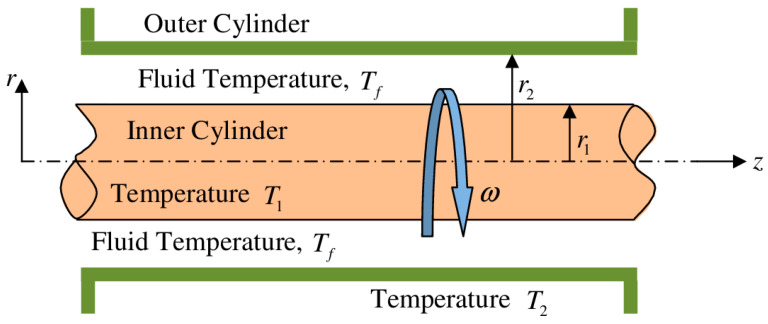
Schematic diagram describing the physical dimensions of the problem. The inner cylinder is rotating at constant angular speed ω. The radii of the inner and outer cylinders are $r_{1}$ and $r_{2}$, respectively. The inner cylinder temperature is $T_{1}$, while the temperature of the outer cylinder is $T_{2}$. Fluid inside the annulus is in motion in the tangential direction as initiated by the rotation of the inner cylinder only.

Here we consider the thermo-fluidic transport (steady) of a Newtonian fluid through an asymmetrically-heated annulus between two concentric cylinders of infinite length, as schematically shown in Figure 1. We assume a circular parallel flow inside the annulus, while the flow is influenced by the rotational movement of the inner cylinder. We do not consider any external pressure gradient to make the flow occur. We consider no-slip boundary conditions at both the walls of the annulus. We also consider thermally developing flow, while for the thermal boundary conditions both the cylinders are considered to be at different (unequal) constant temperatures. Since the length of the annulus is very large compared to the radial gap between two cylinders, we consider $\frac{\partial }{\partial z}\left(\right)\approx 0$ in this analysis. Furthermore, here we consider the flow to be rotational symmetry, which in turn makes $\frac{\partial }{\partial \phi }\left(\right)\approx 0$. In addition to the above, we also consider a few assumptions for the present study as follows:

- steady flow of an incompressible Newtonian fluid;
- inner cylinder is rotating at constant speed;
- there is no heat generation and thermo-physical properties are constant;
- axial conduction is neglected in the fluid and through the wall.

### 2.2. Governing Equations

The fundamental equations governing the thermo-fluidic transport in this study are the incompressible continuity and momentum equation for fluid flow, and the thermal-energy equation for the transport of heat. Since the equations are derived based on the assumptions stated above, the continuity equation, pertinent to the problem considered here, was satisfied automatically. We write the momentum and thermal energy equations as below:

Momentum equation:

(1) $$\frac{d^{2}v_{\phi }}{dr^{2}}+\frac{d}{dr}\left(\frac{v_{\phi }}{r}\right)-\frac{v_{\phi }}{r^{2}}=0$$

The boundary conditions for Equation (1) are given by:

(2) $$\begin{array}{lll} v_{\phi }=\omega r_{1} & \mathrm{at} & r=r_{1}\\ v_{\phi }=0 & \mathrm{at} & r=r_{2} \end{array}\}$$

We obtain the expression of velocity distribution as:

(3) $$v_{\phi }=C_{1}r+\frac{C_{2}}{r}$$

The constants $C_{1}$ and $C_{2}$ appearing in Equation (3) are obtained by utilising the above boundary conditions. Below, we write the explicit expression of these constants as:

(4) $$C_{1}=\frac{\omega {r_{1}}^{2}}{\left({r_{1}}^{2}-{r_{2}}^{2}\right)}\;\mathrm{and}\;C_{2}=\frac{\omega {r_{1}}^{2}{r_{2}}^{2}}{\left({r_{2}}^{2}-{r_{1}}^{2}\right)}$$

Energy equation:

The governing equation for thermal-energy conservation, pertinent to the problem considered in this study, is,

(5) $$k\frac{1}{r}\frac{d}{dr}\left(r\frac{dT}{dr}\right)+\mu {\left(\frac{dv_{\phi }}{dr}-\frac{v_{\phi }}{r}\right)}^{2}=0$$

The boundary conditions for Equation (1) are given by:

$$\begin{array}{ll} T=T_{1} & \;\mathrm{at}\;r=r_{1}\\ T=T_{2} & \;\mathrm{at}\;r=r_{2} \end{array}\}$$

The terms k and μ appearing in Equation 5 are the thermal conductivity and viscosity of the fluid, respectively. It is important to mention here that the convection term for the circular Couette flow, following the assumptions employed here, automatically drop out from the energy equation as apparent from the equation given above. We next proceed to obtain the closed-form expression of temperature distribution, Nusselt number and entropy-generation number.

### 2.3. Analysis Outline: Expression of Temperature Distribution and Nusselt Number

In order to assess the combined influences of viscous heating and the degree of asymmetrical wall heating on the underlying thermal transport, we make an attempt here to solve the energy equation in constant (unequal) wall-temperature thermal-boundary conditions as employed in this study, taking the viscous dissipation term (the second term on the left-hand side of Equation (5)) into account. This essentially tells us how to investigate the closed-form expression of the Nusselt numbers and entropy-generation number in the present analysis. Considering that aspect, we take an effort to solve the thermal-energy equation using an analytical framework.

We first try to write the energy equation, as represented by Equation (5), into its dimensionless equivalent form. However, to express Equation (5) in a non-dimensional framework, we define a few non-dimensional parameters. Note that the dimensionless parameters are chosen from the physical considerations pertinent to the case taken up in the present work and as given by:

(6) $$R=r/r_{1},\;R^{*}=r_{2}/r_{1},\;V=v_{\phi }/\omega r_{1},\;\theta =\left(T-\bar{T}\right)/\left(T_{f}-\bar{T}\right)$$

where Ω is the rotational speed of the inner cylinder. The temperature $T_{f}$ is the uniform fluid inlet temperature and $\bar{T}\left(=\left(T_{1}+T_{2}/2\right)\right)$ is the average wall temperature. To take the effect of unequal wall temperature into account, we define a parameter as characterized by the degree of asymmetrical wall heating. This asymmetrical wall heating parameter is symbolized by $\beta =\left(T_{2}-T_{f}\right)/\left(T_{1}-T_{f}\right)$ [24,25].

However, with the aid of the above dimensionless parameters and using Equation (3), we normalize Equation (5) to yield the following equation:

(7) $$\frac{k\left(T_{f}-\bar{T}\right)}{R}\frac{d}{dR}\left(R\frac{d\theta }{dR}\right)+\mu {\left(\omega r_{1}\right)}^{2}\;{\left(\frac{dV}{dR}-\frac{V}{R}\right)}^{2}=0$$

We now write the expression of the velocity distribution in the non-dimensional form as below:

(8) $$V=B\left(R-\frac{R^{*}{}^{2}}{R^{2}}\right)$$

where the term B is defined as: $B=1/\left(1-R^{{*}^{2}}\right)$. Now using Equation (8), we recast (7) in the following form as:

(9) $$\frac{1}{R}\frac{d}{dR}\left(R\frac{d\theta }{dR}\right)+4Br\frac{P}{R^{4}}=0$$

Note that, in arriving at Equation (9), we introduce the Brinkman number Br [26] and a constant P (introduced for the sake of the analysis), which are defined as:

(10) $$Br=\frac{\mu {\left(\omega r_{1}\right)}^{2}}{k\left(T_{f}-\bar{T}\right)}\;\mathrm{and}\;P=\frac{R^{*}{}^{4}}{{\left[R^{*}{}^{2}-1\right]}^{2}}$$

In order to obtain the temperature profile, we solve Equation (9) using the thermal-boundary conditions as given above. However, in a non-dimensional form, the above set of boundary conditions may be expressed as:

(11) $$\begin{array}{ll} \theta =\left(T_{1}-\bar{T}\right)/\left(T_{f}-\bar{T}\right) & \;\mathrm{at}\;R=1\\ \theta =\left(T_{2}-\bar{T}\right)/\left(T_{f}-\bar{T}\right) & \;\mathrm{at}\;R=R^{*} \end{array}\}$$

We further simplify Equation (9) using the boundary conditions as given in Equation (11) to yield the general expression of the temperature profile, as below:

(12) $$\theta =BrP\left[1-\frac{1}{R^{2}}+\left(\frac{1}{R^{*}{}^{2}}-1\right)\frac{\ln R}{\ln R^{*}}\right]+D\left(1-\frac{\ln R}{\ln R^{*}}\right)$$

Note that in arriving at Equation (10), we introduce another parameter D(=(1−β)/(1+β)), where β is the degree of asymmetrical parameter as defined above.

The main physical quantity of practical interest is the Nusselt number, which gives an estimate about the thermal transport characteristics of heat in the flow field. Thus, here we investigate the explicit expression of the Nusselt numbers (both at the inner and outer cylinders) using the temperature distribution in the flow field as delineated above in Equation (12).

In order to obtain deeper insights into the heat-transfer characteristics, we further define the bulk mean fluid temperature $T_{m}$, which for the present problem can be written as:

(13) $$T_{m}=\int_{r_{1}}^{r_{2}}v_{\phi }Trdr/\int_{r_{1}}^{r_{2}}v_{\phi }rdr$$

Therefore, the heat transfer at the inner cylinder is:

(14) $$q_{1}=-k{\frac{\partial T}{\partial r}|}_{r=r_{1}}=h_{1}\left(T_{1}-T_{m}\right)$$

where $h_{1}$ is the convective heat transfer coefficient. So, the expression of the Nusselt number at the inner cylinder comes out as:

(15) $$Nu_{1}=\frac{h_{1}\left(r_{2}-r_{1}\right)}{k}=\left[\left(R^{*}-1\right)/\left(\theta_{m}-\theta_{1}\right)\right]{\left(\frac{\partial \theta }{\partial R}\right)|}_{R=1}$$

In a similar fashion, we evaluate the expression of the Nusselt number at the outer cylinder and given as:

(16) $$Nu_{2}=\frac{h_{2}\left(r_{2}-r_{1}\right)}{k}=\left[\left(R^{*}-1\right)/\left(\theta_{2}-\theta_{m}\right)\right]{\left(\frac{\partial \theta }{\partial R}\right)|}_{R=R^{*}}$$

where, $\theta_{m}$ is the non-dimensional form of the bulk mean temperature, and $\theta_{1}$, $\theta_{2}$ are the non-dimensional temperatures at the inner and outer cylinder, respectively.

We next make an attempt to obtain the expression of $\theta_{m}$, which will, in turn, helps us in obtaining the closed-form expression of the limiting Nusselt numbers at both the walls of the annulus. However, Equation (13) may be normalized to yield the non-dimensional expression of the mean temperature as:

(17) $$\theta_{m}=\left(T_{m}-\bar{T}\right)/\left(T_{f}-\bar{T}\right)=\int_{1}^{R^{*}}V\theta RdR/\int_{1}^{R^{*}}VRdR$$

Also, the expressions of non-dimensional temperature at both the walls are as follows:

(18) $$\begin{array}{l} \theta_{1}=-D\\ \theta_{2}=D \end{array}\}$$

We now make use of Equations (15), (17) and (18) to obtain an explicit expression of the Nusselt number at the inner cylinder, which gives the quantitative relation among different physical parameters governing the thermo-fluidic transport for the problem under consideration and is written as:

(19) $$Nu_{1}=\left[\left(R^{*}-1\right)\left\{2BrP+\frac{\left\{BrP\left\{\left(1/R^{*}{}^{2}\right)-1\right\}-2D\right\}}{\ln\left(R^{*}\right)}\right\}\right]{\left\{\theta_{m}+\frac{\left(1-\beta \right)}{\left(1+\beta \right)}\right\}}^{-1}$$

Proceeding in a similar way we evaluate, using Equations (16)–(18), the explicit expression of the Nusselt number at the outer cylinder as follows:

(20) $$Nu_{2}=\left[\left(R^{*}-1\right)\left\{\frac{2BrP}{R^{*}{}^{3}}+\frac{\left\{BrP\left\{\left(1/R^{*}{}^{2}\right)-1\right\}-2D\right\}}{R^{*}\ln\left(R^{*}\right)}\right\}\right]{\left\{\frac{\left(1-\beta \right)}{\left(1+\beta \right)}-\theta_{m}\right\}}^{-1}$$

Below, we write the expression of the dimensionless mean temperature $\theta_{m}$ as:

(21) $$\theta_{m}=\frac{\left(I_{1}+I_{2}+I_{3}\right)}{I_{4}}$$

Each term i.e., $I_{1}-I_{4}$ in Equation (21), is a function of $R^{*}$ and other parameters defined above, as given below:

(22) $$\begin{array}{l} I_{1}=BD\left(R^{*}{}^{2}-\frac{1}{3}-\frac{2}{3}R^{*}{}^{3}\right)\\ I_{2}=\frac{2BrPB}{3}{\left(1-R^{*}\right)}^{3}\\ I_{3}=\frac{\left\{BrPB\left(\frac{1}{R^{*}{}^{2}}-1\right)-2BD\right\}}{9\ln\left(R^{*}\right)}\left\{1+2R^{*}{}^{3}\left(4-3\ln R^{*}\right)-9R^{*}{}^{2}\right\}\\ I_{4}=BR^{*}{}^{2}\left(1-\frac{2}{3}R^{*}\right)-\frac{B}{3} \end{array}\}$$

Based on the derivations mentioned above, the heat-transfer characteristics in a viscous dissipative flow environment can be investigated. We obtain the expression of the entropy generation in the annulus in the next section.

### 2.4. Entropy Generation in the Annulus

When fluid flows over the heated surface for convective transport heat, a spontaneous exchange of momentum and energy gives rise to entropy generation following the thermodynamic irreversibility in the system. Viscous heating plays an important role in inviting irreversibility into the thermodynamic process. In the context of the present study, irreversibility arises from two different sources, viz., the heat transfer between the fluid and solid walls, and the viscous heating, which is inevitable in the course of fluid motion initiated by the movement of the solid boundary. Below, we write the general expression for the volumetric entropy-generation rate pertinent to the problem as given by [1,3,27]:

(23) $$S_{G}=\frac{k}{T_{f}^{2}}{\left(\frac{dT}{dr}\right)}^{2}+\frac{\mu }{T_{f}}\Phi$$

where Φ is the viscous-dissipation term. The first term in Equation (17) denotes the irreversibility due to heat transfer, while the second term Φ indicates the fluid-friction dominant irreversibility. The viscous-dissipation function pertinent to the present problem as considered here is given by,

(24) $$\Phi ={\left(\frac{dv_{\theta }}{dr}-\frac{v_{\theta }}{r}\right)}^{2}$$

We then write the dimensionless entropy-generation number using the non-dimensional parameters as mentioned in Section 2.3. However, the dimensionless entropy-generation number can be written as [1,27]:

(25) $$N_{S}={\left(\frac{d\theta }{dR}\right)}^{2}+Br\Omega^{-1}{\left(\frac{dV}{dR}-\frac{V}{R}\right)}^{2}$$

where $\Omega \left(=\left(T_{f}-\bar{T}\right)/T_{f}\right)$ is the temperature difference parameter and $Br\Omega^{-1}$ is defined as the group parameter. Note that the first and second terms of Equation (18) represent the irreversibility associated with the heat transfer ($N_{HT}$) and fluid friction ($N_{FF}$), respectively. One may obtain, using Equations (8), (12) and (25), the total entropy generation for the present problem. Since the entropy-generation number $N_{s}$ as given above in Equation (25) takes both the heat transfer and fluid-friction dominant irreversibility into account, it is imperative to develop an understanding about the dominating role of either of the above terms in contributing the total entropy-generation rate in the system as considered in this study. In order to do so, we appeal to the concept of the irreversibility distribution ratio and defined as: $\phi =N_{FF}/N_{HT}$ [1]. From the expression of ϕ, one can appreciate that, for 0≤ϕ≤1, the heat-transfer influenced entropy generation takes a lead role in governing the total entropy of the system, while for ϕ>1, the effect of fluid friction becomes crucial in generating thermodynamic irreversibility in the system. A clear understanding about the entropy generation due to heat transfer $\left(N_{HT}\right)$ to the total entropy generation $\left(N_{s}\right)$ in any process or system involved with thermo-fluidic transport is crucial for their optimum design, which allows these systems to be operated at their maximum exergetic efficiency. In precise terms, to express such an irreversibility-distribution ratio in any thermodynamics process, a dimensionless number, which is called as the Bejan number Be, is introduced and mathematically expressed as [1,3,27]:

(26) $$Be=N_{HT}/N_{S}=1/\left(1+\phi \right)$$

Note from Equation (2) that that the Bejan number takes two extreme limits, “0” and “1”. The upper limit “1” indicates that the heat-transfer influenced irreversibility dominating over the irreversibility that arises due to the viscous dissipation effect, while the lower limit “0” corresponds to the viscous dissipation dominant irreversibility in the system.

## 3. Results and Discussions

Since the present analysis aims to find out the effect of viscous heating as produced by the rotation of the inner cylinder on the underlying thermal transport in an asymmetrical heating environment, we vary the temperature distribution, Nusselt number and the entropy-generation number under different conditions. In this study we have depicted the variation of all numbers and the temperature for different values Br and β. We have clearly specified the different parameters considered in plotting a figure in the respective figure caption as well as in the text. However, we consider Br=±0.1 and β=±0.5, which are typically used in the literature [7,8,15,24,28]. The Brinkman number is defined in this study as follows: $Br=\frac{\mu {\left(\omega r_{1}\right)}^{2}}{k\left(T_{f}-\bar{T}\right)}$. If we assume water is used as a fluid in this analysis, and the change in temperature $\left(T_{f}-\bar{T}\right)∼10K$, then the typical value of Brinkman number, for μ=0.001 Pa⋅s, k=0.6 W/mK, ω=100 RPM and $r_{1}=10\;\mathrm{cm}$, becomes $Br∼10^{-2}-10^{-1}$. Moreover, we have considered $R^{*}(=r_{2}/r_{1})$ in the range of 1.2 to 2. Thus, for $r_{1}=10\;\mathrm{cm}$, the minimum gap of the annulus becomes ~2000 μm. However, in the present study, we have considered a higher value of $Br∼10^{-1}$ to investigate the effect of viscous dissipation on the underlying thermo-hydrodynamics.

### 3.1. Variation of Temperature in the Flow Field

**Figure 2:**
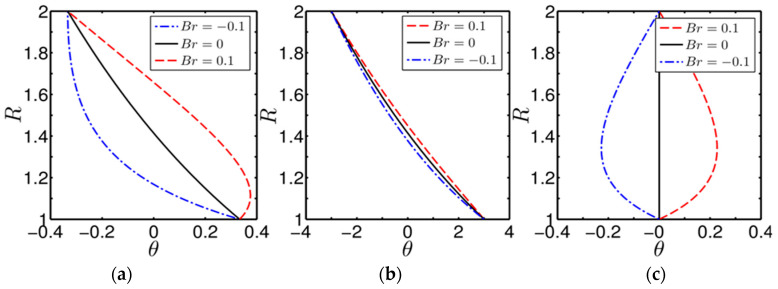
Plot showing the variation of dimensionless temperature distribution in the flow field for different values of *Br* and obtained in different cases of wall heating: (**a**) for β=0.5; (**b**) for β=−0.5; and (**c**) for β=1.

Figure 2a–c shows the variation of temperature distribution inside the annulus for different degrees of asymmetrical wall heating paremeter (β). Figure 2a–c corresponds to β=0.5, −0.5 and 1, respectively. Note that β=1 indicates a symmetrical heating condition in the present scenario. We have considered β=1 in this analysis only to demarcate the effect of thermal asymmetry on the thermal transport characteristics of heat as manifested clearly by the variation of temperature distribution in the flow field.

For each case of wall heating, we have considered three different values of Br(=0.1,−0.1 and 0) to obtain the temperature profile as influenced by the shear-heating generated by the rotating cylinder. We should mention here that viscous dissipation always generates a distribution of heat source and stimulates the internal energy in the fluid, which in turn distorts the temperature profile as envisaged from the figures under present consideration. In fact, a strong influence of the viscous heating effect on the temperature distribution in the flow field is supported by an observation that, in cases with Br≠0, the profile of the dimensionless temperature is altered in comparison with the case having Br=0, although the imposed boundary condition on the walls remains invariant. In the thermal entrance region, a linear trend of developing dimensionless temperature θ is observed for all the cases of wall heating with Br=0, which is a pure conduction profile. It can be observed from the figures that the nonexistence of viscous dissipation makes the temperature distribution independent, irrespective of the asymmetry of the wall heating considered in the analysis. Two points can be made about Figure 2 as follows: first, positive values of Br are compatible with the wall-heating case, which resembles the situation of heat transfer to the fluid across the wall. Therefore, for cases with positive values of Br, the fluid temperature is increased, as seen from Figure 2a–c. On the contrary, negative values of Br represent the wall-cooling case and weaken the fluid temperature by transferring heat from the fluid to the wall. We can confirm the reduction in fluid temperature for negative Br in the present figures as well. Second, the fluid temperature, for positive Br, in the region closer to the inner cylinder shows an increasing trend for β=0.5 and 1, respectively, which one may see from Figure 2a,c. In this context, the rotation of the inner cylinder deforms the surrounding fluid layer, leading to the development of shear heating in that region. We mimic the rotation-induced development of shear heating by the positive values of Br. Therefore, shear heating, stemming from the rotational effect of the inner cylinder, heats up the fluid in that region and results in an increase in the fluid temperature as clearly reflected in Figure 2a,c. Having a closer look at Figure 2a–c, we also observe a quantitative change in the fluid temperature with a change in β, albeit the parameter Br remains unaltered. Also, it is worth noting from Figure 2c that the variation of temperature with Br becomes perfectly symmetrical. We attribute these two observations to the the effect of the degree of the asymmetrical-heating parameter on the thermal-transport characteristics of heat. However, the variations in the temperature profile with different values of Brinkman number, as observed in the above figures, are in tune with what it is expected and show similarity with reported results [7,28,29].

**Figure 3:**
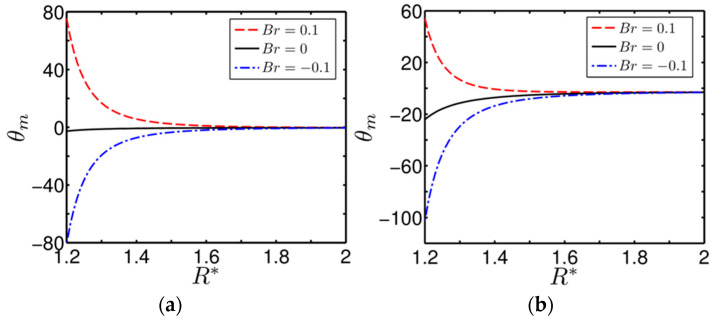
Plot of the variation of dimensionless bulk-mean temperature $\theta_{m}$ versus $R^{*}$ (aspect ratio of the annulus) for different values of *Br* and obtained at two different degrees of asymmetrical wall heating parameter: (**a**) β=0.5 and (**b**) β=−0.5.

The temperature inside the annulus is highly non-uniform, with this non-uniformity stemming from two important effects: one is the effect of thermal asymmetry, and the second is the effect of the continuous rotation of the inner cylinder. Taking these two important issues into account, we calculate the mean temperature for a fair assessment about the heat transfer rate at the walls of the annulus. Accordingly, we show in Figure 3a,b the variation of bulk mean temperature $\theta_{m}$ versus $R^{*}$ (aspect ratio of the annulus) in the annulus for different values of Br, obtained for two different degrees of asymmetrical heating β=0.5 and −0.5, respectively. However, one may note from the above figures that the profiles of the dimensionless bulk mean temperature with $R^{*}$ are strongly dependent on the degree of asymmetry in wall heating β. Since at $R^{*}=1$, the solution becomes undefined (the effect of the point of singularity), we vary $R^{*}$ in the range $1.2<R^{*}<2$, while plotting Figure 3a,b as delineated above. We observe that the mean temperature for Br=0.1 becomes almost positive in the annulus, whereas it becomes negative for Br=−0.1. The behaviour of the mean temperature as seen in Figure 3a,b is expected, since positive Br mimics a situation of heating up the fluid, and so increases the mean temperature. By contrast, negative Br weakens the fluid temperature, thus resulting in a negative mean temperature, as clearly reflected in Figure 3b. One may also note from Figure 3a,b that the variation of mean temperature with $R^{*}$, in the region closer to the inner cylinder, exhibits a step gradient. This observation once again signifies the effect of shear heating developed due to the rotational effect of the cylinder on the underlying thermal transport. It is interesting to observe from Figure 3a,b that, for any non-zero values of Br, the variation of $\theta_{m}$ with $R^{*}$ obtained at β=0.5 are symmetrical about the profile of the same order obtained at Br=0, while we observe an asymmetrical variation for β=−0.5. Moreover, one can clearly see from Figure 3a,b that the variation of $\theta_{m}$ with $R^{*}$, for all degrees of thermal asymmetry considered, becomes independent of Br near the outer cylinder. We attribute these observations to the inequality of the wall temperature of the annulus together with the effect of viscous heating induced by the rotation of the inner cylinder.

### 3.2. Variation of the Nusselt Number

**Figure 4:**
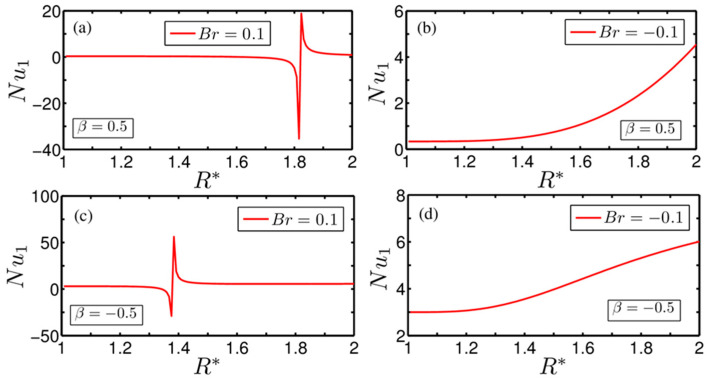
Plot showing the influence of Br on the variation of the inner wall Nusselt number $Nu_{1}$ obtained at two different values of asymmetrical wall heating β=0.5 and −0.5, respectively. Two different Br=0.1 and −0.1 have been considered for the above plots. The combination of different parameters chosen for the plotting are: (**a**) Br=0.1 and β=0.5; (**b**) Br=−0.1 and β=0.5; (**c**) Br=0.1 and β=−0.5; and (**d**) Br=−0.1 and β=−0.5.

Here we discuss variation of the Nusselt number, which gives the quantitative estimate of the heat transfer rate at the walls of the annulus, for the different physical parameters considered in this study. We depict in Figure 4a–d the variation of the Nusselt number at the inner cylinder graphically for two different values of the Brinkman number Br=±0.1, as considered in this study. Note that Figure 4a,b correspond to the cases of asymmetric wall heating β=0.5, while in Figure 4c,d we consider β=−0.5 to obtain the desired variation. Equations (19) and (20) represent the expression of the Nusselt number on the inner cylinder and outer cylinder, respectively. One may clearly observe from Equations (19) and (20) that both Nusselt numbers are functions of two independent variables, e.g., the degree of asymmetry in the wall heating β and the Brinkman number Br. However, both the Nusselt numbers will have parametric variation with Br for Br≠0 and with β for β≠1.

Note that for Br=0.1, the variation of $Nu_{1}$ with $R^{*}$ is not continuous for all cases of the degree of asymmetry considered in the study (see Figure 4a,c); rather, for each case the variation of the Nusselt number exhibits a discontinuity in behaviour, leading to a point of singularity at different $R^{*}$. As mentioned, the positive Br represents a wall-heating case, which mimics a situation of transferring heat from the wall to the fluid. It needs to be mentioned here that shear heating generated by the rotational effect of the inner cylinder increases the fluid temperature too. Since the fluid temperature, in the region closer to the inner cylinder increases owing to induced viscous heating, the variation of $Nu_{1}$ in this region becomes flat, signifying no driving temperature difference for heat transfer. Thus, for a positive value of Br, the system encounters a situation where the temperature within the fluid becomes equal to the wall temperature at that prevailing thermal-boundary condition, which in turn gives rise to an unbounded swing, as apparent from Figure 4a,c. Careful observation of Figure 4a,c reveals that the location of the onset of the point of singularity changes with the alteration in the degree of asymmetrical wall heating, largely attributed to the thermal condition (temperature) of the wall. We should mention here that the onset of the point of singularity is an outcome of the local equilibrium of energy wherein the internal heat generation due to viscous dissipation balances the heat supplied by the wall. A closer scrutiny of Figure 2a confirms the appearance of inflections on the variation of the temperature profile specific to the case of Br=±0.1 and β=0.5. To be precise, the temperature profile exhibits global maxima and global minima for Br>0 and Br<0, respectively, and at this point the supplied heat from the wall and the dissipative heat generated due to fluid friction become equal, causing no heat to flow in either direction and leading to the appearance of an unbounded swing, as seen in Figure 4a,c.

By contrast, we observe an increasing trend on the variation of $Nu_{1}$ for Br=−0.1 even for all the cases of asymmetrical heating (see Figure 4b,d). A negative value of Br resembles a situation of wall cooling, i.e., transferring heat from the fluid to the wall. So an increasing trend of $Nu_{1}$ for a negative value of Br, as seen from Figure 4b,d, once more signifies the effect of viscous heating generated by the rotational effect of the inner cylinder.

**Figure 5:**
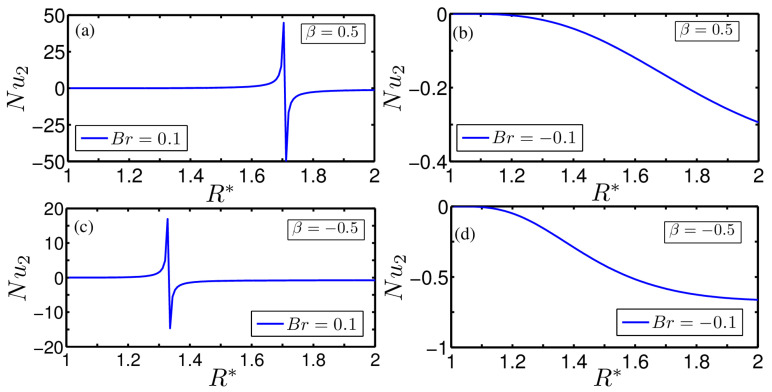
Plot showing the influence of Br on the variation of the outer wall Nusselt number $Nu_{2}$ obtained at two different values of asymmetrical wall heating β=0.5 and −0.5, respectively. Two different Br=0.1 and −0.1 have been considered for the above plots. The combination of different parameters chosen for the plotting are: (**a**) Br=0.1 and β=0.5; (**b**) Br=−0.1 and β=0.5; (**c**) Br=0.1 and β=−0.5; and (**d**) Br=−0.1 and β=−0.5.

Figure 5a–d show the influence of Br on the variation of the Nusselt number at the outer wall of the annulus $Nu_{2}$ with $R^{*}$ for different degrees of asymmetrical wall heating β=0.5 and −0.5, respectively. We still observe the point of singularity on the variation of $Nu_{2}$ for positive Br irrespective of β. A closer look at Figure 5a,c reveals that the location of the onset of the point of singularity on the variation of $Nu_{2}$ is different from those appearing on the variation of $Nu_{1}$, primarily attributed to the effect of viscous heating as induced by the inner cylinder rotation. For both cases of wall heating, one may observe an increasing trend of $Nu_{2}$ with $R^{*}$ for Br=−0.1, as apparent from Figure 5b,d. We can also observe from Figure 5b,d that the rate of heat transfer at the outer wall is more for β=−0.5, signifying the effect of thermal asymmetries on the underlying transport of heat. From the point of singularity, both the Nusselt numbers decrease in both directions and asymptotically reach at constant value at both the inner and outer walls of the annulus. An important observation can be made from Figure 4 and Figure 5 that, for the cases with β≠1, the value of the Nusselt number on both the walls of the annulus does not match as $R^{*}\to 1$. This is primarily attributable to the rotation of the inner cylinder, which enhances the temperature of the fluid layer in the region close to the inner wall following a significant contribution to the viscous dissipation effect. The physical explanation for the negative Nusselt number, as seen from the figures presented above, stems from the appearance of inflections on the temperature profile, where the cross-sectional averaged fluid temperature becomes larger than the temperature imposed at the wall and hence heat transfer takes place in the reverse direction.

**Figure 6:**
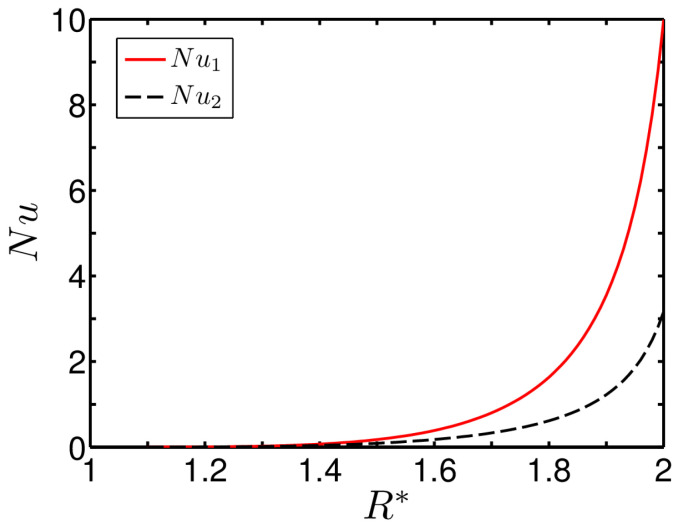
Plot of the variation of the Nusselt number versus $R^{*}$. The other parameters considered for this plot are Br=0.1 and β=1.

It can be seen that the rotation of the inner cylinder imposes additional shear stress on the fluid, which aggravates the viscous heating resulting in reduction in the heat transfer at the inner wall of the annulus. Additionally, from the figures presented above, one may find that the dependence of Nusselt numbers on Br, $R^{*}$ and β is important for β≠1. In an effort to bring out the effect of β on the heat-transfer rate, we depict in Figure 6 the variation of both the Nusselt numbers for the case of symmetrical wall heating β=1. We consider Br=0.1 in plotting Figure 6.

It is important to observe from Figure 6 that, in the region closer to the outer wall of the annulus, $Nu_{1}$ is significantly larger than $Nu_{2}$ even in a symmetrical wall-heating scenario. Such behaviour of the Nusselt number can be explained from the induced viscous-heating effect stemming from the rotation of the inner cylinder. The induced viscous heat increases the surrounding fluid temperature in the region closer to the inner wall of the annulus. On the other hand, away from the inner wall, the rotational effect of the inner cylinder is not felt so severely, leading to a relatively lower fluid temperature. Since the fluid temperature in the zone closer to the outer wall of the annulus is relatively lower, we find an augmentation in the heat-transfer rate due to the increased driving potential of temperature difference, as clearly reflected in Figure 6.

Here we discuss the appearance of point of singularity on the variation of the Nusselt number from the second law of thermodynamics. In the context of micro-scale convective heat transport analysis, there could be a situation where the dissipative heat arising even for a small Br(≪1) becomes important, since the viscous heat significantly alters the heat-transfer characteristics over small scales [11,12,13,14]. It is imperative to mention that all thermodynamic devices working with any system should ensure the maximum possible heat transfer from the processes involved in order to increase system efficiency. By contrast, an enhancement in the rate of heat transfer imposes a constraint on the same as far as the thermodynamic irreversibility of the system is concerned, since an increased heat-transfer rate increases the system irreversibility as well. This contradictory behavior of heat-transfer enhancement with the increased rate of entropy generation makes it obligatory for the thermal systems/devices to compromise on the viscous dissipation dominant irreversibility in the process. To be precise, the onset of the point of singularity on the variation of the Nusselt number is essentially a reflection of such a minimization process, as discussed in the next paragraph.

In this analysis two different degrees of asymmetrical wall heating have been taken into account to obtain the variation of both the Nusselt numbers evident from the figures portrayed above. The irreversibility associated with the heat-transfer rate for two different degrees of asymmetry parameter, however, compromises on the viscous heating dominant irreversibility in the system to obtain the maximum possible heat-transfer rate in the process. However, this process of the minimization of viscous heating dominant irreversibility of the system as considered in the present study leads to the onset of the point of singularity, as observed by the variation of the Nusselt number for both the cases of asymmetrical wall heating. 

### 3.3. Thermodynamic Irreversibility Analysis: Variation of the Entropy-Generation Number and the Bejan Number

**Figure 7:**
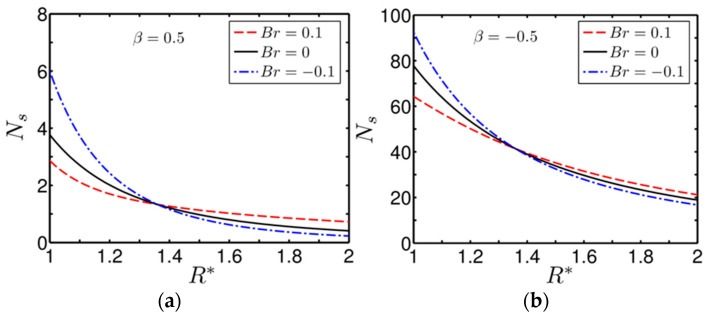
Plot showing the variation of the entropy-generation number $\left(N_{s}\right)$ for different values of Br for two different cases of asymmetrical wall heating: (**a**) β=0.5; and (**b**) β=−0.5. The other parameter considered is $Br\Omega^{-1}=0.4$.

We show the variation of the volumetric entropy-generation number versus $R^{*}$ in Figure 7a,b for different values of Br(=±0.1,0), as considered in this study. Figure 7a,b correspond to the degree of asymmetrical parameter β=0.5 and −0.5, respectively. We also consider the following parameter in plotting the figures as: $Br\Omega^{-1}=0.4$. One may see from Figure 7a,b that, for both the cases of wall heating, entropy generation at the inner wall of the annulus is higher compared to that of the outer wall. We also find that the entropy generation at the inner wall of the annulus becomes maximum and minimum for Br=−0.1 and −0.1, respectively. By contrast, a reverse phenomenon occurs at the outer wall of the annulus. In fact, these observations hold true for both wall-heating scenarios, as apparent from Figure 7a,b.

A positive Br represents a wall-heating case, which indicates that heat is being transferred from the wall to the fluid. Since the rotation of the inner cylinder induces viscous heating in the surrounding fluid layer, the heat-transfer rate is reduced in that region owing to the diminished strength of the driving temperature difference potential. A relatively lesser heat transfer rate, intrinsic to Br=0.1, reduces the entropy-generation rate in the region of the inner cylinder for both cases of asymmetrical wall heating, as seen from Figure 7a,b. On the other hand, since negative Br(=−0.1) represents the wall-cooling case, heat transfer is enhanced in the region closer to the inner cylinder owing to the increasing strength of the driving temperature difference, which in turn culminates in an enhancement of the entropy-generation rate. We confirm this phenomenon in Figure 7a,b as well. By contrast, we can observe a reverse scenario at the outer wall of the annulus and its surrounding zone. At the outer wall of the annulus, no source contributes viscous heating to the fluid, except that which arises due to the frictional effect of fluid layers; thus, the transfer and so the entropy-generation rate become higher for positive Br (since for positive Br the driving temperature difference for heat transfer becomes higher) for both the cases of wall heating. It is worth mentioning here that such behaviour of the entropy generation rate $\left(N_{s}\right)$ with Br inside the annulus gives rise to a distinct crossover at some $R^{*}$, as clearly visible from the figures being considered. However, closer scrutiny of Figure 7a,b reveals a quantitative distinction in the entropy-generation rate inside the annulus for varying degrees of asymmetrical heating β, largely attributed to the heat-transfer rate. From the above discussion, it may be inferred that the effect of viscous heating takes a lead role in contributing the total entropy-generation rate in the inner wall of the annulus, while heat transfer influences the entropy generation rate surrounding the outer wall of the annulus considerably. We will discuss the relative contribution of the two important factors viz., the viscous dissipation effect and heat transfer on the total entropy-generation of the system being considered in the next section.

**Figure 8:**
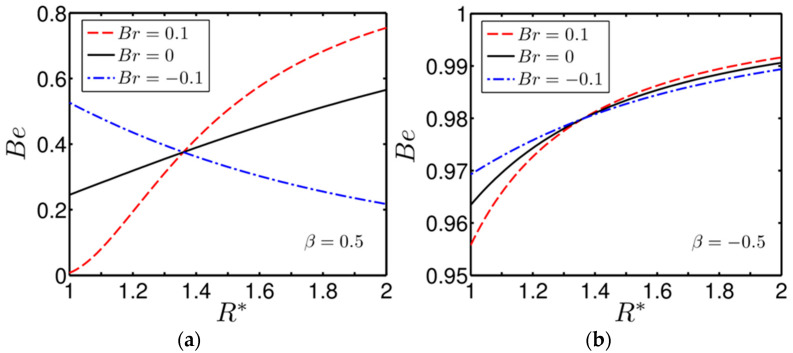
Plot of the variation of the Bejan number (Be) for different values of Br, obtained at two different cases of asymmetrical wall heating: (**a**) β=0.5; and (**b**) β=−0.5. The other parameter considered is $Br\Omega^{-1}=0.4$.

Figure 8a,b depicts variation of the Bejan number (Be) with $R^{*}$ for two different cases of asymmetrical wall heating β=0.5 and −0.5, respectively. While plotting the Bejan number, we consider three different values of Br(=±0.1,0) to see the effect of viscous dissipation on the irreversibility-generation behavior. Note that all the values of Br chosen in depicting the present figures (Figure 8a,b) are in compliance with those considered in Figure 7a,b. One can see that in the region closer to the inner wall of the annulus, viscous heating plays a dominating role over the heat transfer in governing the irreversibility for Br=0.1, while the effect of heat transfer become crucial in contributing the thermodynamic irreversibility for Br=−0.1. This observation is true for both cases of wall heating, as evident from Figure 8a,b. The observations reflected in Figure 8a,b are in clear support of the behaviour of the entropy-generation rate as delineated in Figure 7a,b.

**Figure 9:**
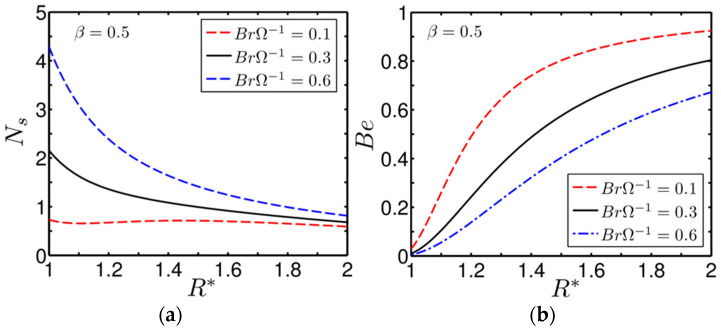
(**a**) Variation of the entropy-generation number for different values of the group parameter; and (**b**) variation of the Bejan number for different values of the group parameter. The other parameters considered are: β=0.5 and Br=0.1.

Figure 9a,b shows the variation of the entropy-generation rate inside the annulus as influenced by the group parameter $Br\Omega^{-1}$. In plotting Figure 9a,b, we consider three different values of the group parameter $Br\Omega^{-1}=0.1,0.3\;\mathrm{and}\;0.6$, respectively. The other parameters considered for plotting the figures are Br=0.1 and β=0.5. Note the group parameter is an important parameter, which signifies the ratio of the effect of viscous heating and thermal asymmetry. The values of the group parameter chosen in depicting the present figure are those typically used in the literature [24,30].

A closer look at Figure 9a reveals that the entropy generation number shows a continual decreasing trend with $R^{*}$ for $Br\Omega^{-1}=0.3\;\mathrm{and}\;0.6$, while the variation becomes almost flat for $Br\Omega^{-1}=0.1$. We can also observe from above figure that, in the inner wall and its surrounding region, the entropy-generation number is higher for a relatively higher value of the group parameter. An increase in the value of $Br\Omega^{-1}$, for a given Br (Br=0.1), indicates a decrease in Ω. Therefore, with an increasing value of $Br\Omega^{-1}$ the heat-transfer rate at both the walls of the annulus drops for a given degree of asymmetrical parameter, which in turn lessens the heat-transfer dominant irreversibility in the system as well. Thus, the increasing rate of entropy generation in the region closer to the inner wall as seen from Figure 9a is largely attributed to the effect of the viscous heating developed due to the rotational effect of the cylinder. We confirm this in Figure 9b as well, where the Bejan number (Be) is seen to be almost zero for $Br\Omega^{-1}=0.6$, signifying the leading role of the viscous-heating effect in inviting total irreversibility into the system. However, with increasing $R^{*}$, the rotation-induced frictional effect decreases, which in turn decreases the entropy-generation rate, as confirmed in Figure 9a as well. In precise terms, at the outer wall and its surrounding region heat transfer takes a lead role in inviting irreversibility, as largely confirmed in Figure 9b, where the Bejan number becomes closer to one. 

## 4. Conclusions

In the present study, we have investigated the forced convective heat-transfer characteristics of a Newtonian fluid through an asymmetrically heated annulus between two concentric cylinders, where the inner cylinder is rotating at constant speed. Using an analytical methodology, we have devised here the closed-form expression of the temperature profile inside the annulus, which in turn gives rise to the explicit and closed-form expression of the Nusselt number as well as the entropy-generation number. We observe that two important factors, the viscous-dissipation effect and the asymmetrical wall-heating parameter, play important roles on the underlying thermal-transport characteristics of heat, which in turn affects the entropy-generation rate in the system significantly. We conclude the following from the analysis:

- The dimensionless temperature profile for positive values of Br shows an increasing trend. On the other hand, the temperature profile for negative values of Br exhibits a reverse trend.
- Specific to the case of a positive value of Br, the Nusselt number at both the walls of the annulus exhibits a discontinuity in behaviour for all the cases of wall-heating considered, leading to a point of singularity at some $R^{*}$. The onset of the point of singular is an outcome of the local energy balance, where heat generated by the viscous dissipation, arising due to the rotational effect of the inner cylinder, balances the heat supplied by the wall. However, the location of the onset of the point of singularity alters with a change in β. This is attributable to alterations in the wall-heating effect arising from different degrees of thermal asymmetry.
- For the negative value of Br, the Nusselt numbers show an increasing trend for both the cases of asymmetrical wall heating. However, the heat-transfer rate is relatively higher for β=−0.5, signifying the effect of the asymmetrical wall heating on the thermal transport of heat.
- The values of the Nusselt number at both the walls of the annulus do not match as $R^{*}\to 2$ even for a symmetric wall heating, i.e., for β≠1.0. This is because of the movement of the inner cylinder, which provides additional shear stress in the fluid culminating in the development of viscous heating. The development of viscous heating results in a drastic change in the overall heat balance in the flow field, which in turn alters the heat-transfer characteristics at the inner wall of the annulus.
- The rotation of the inner cylinder induces viscous heating in the surrounding fluid layer, which in turn reduces the heat-transfer rate in that region owing to the diminished strength of the driving temperature-difference potential. Thus, a positive Br(=0.1), which mimics a situation of heat transfer from the wall to the fluid, reduces the entropy-generation rate in the region of the inner cylinder for both the cases of asymmetrical wall heating following the effect of a lesser heat-transfer rate from the wall to the fluid. By contrast, the heat-transfer rate for a negative Br(=−0.1) is higher at the inner cylinder, leading to relatively higher entropy generation there. We observe a reverse trend of entropy generation at the outer wall of the annulus for Br.
- We also make an effort to calculate the Bejan number for different values of Br in two different cases of asymmetrical wall heating. The variation of the Bejan number clearly supports the behaviour of the entropy-generation rate obtained for different cases.

We believe that inferences obtained from the present analysis may provide some insights about the thermal-transport characterstics of heat and its effects on the entropy-generation rate in micro-rotataing devices/systems that have so far been unexplored.

## Nomenclature

B	Parameter to characterize $\left[1/\left(1-R^{{*}^{2}}\right)\right]$
Be	Bejan number
Br	Brinkmann number
$Br\Omega^{-1}$	group parameter
$C_{1}-C_{2}$	Constants
$c_{p}$	Specific heat at constant pressure (J/g K)
D	Parameter to characterize (1−β)/(1+β)
*h*_1_	heat-transfer coefficient at inner wall (W/m^2^·K)
*h*_2_	heat-transfer coefficient at outer wall (W/m^2^·K)
$I_{1}-I_{4}$	constants, depends on aspect ratio
k	thermal conductivity (W/m·K)
$N_{s}$	dimensionless entropy-generation number
$N_{HT}$	entropy generation due to heat transfer
$N_{FF}$	entropy generation due to fluid friction
*Nu*	Nusselt number
*Nu*_1_	Nusselt number at the inner wall of the annulus
*Nu*_2_	Nusselt number at the outer wall of the annulus
P	Parameter to characterize ${\left[R^{*}{}^{2}/\left(1-R^{{*}^{2}}\right)\right]}^{2}$
*q*_1_	lower wall heat flux (W/m^2^)
*q*_2_	upper wall heat flux (W/m^2^)
r	radial coordinate (m)
$r_{1}$	inner radius of the annulus (m)
$r_{2}$	outer radius of the annulus (m)
R	Dimensionless radial coordinate
$R^{*}$	aspect ratio of the annulus
$S_{G}$	volumetric entropy generation (W/m^3^·K)
T	temperature (K)
$\bar{T}$	average temperature (K)
$T_{1}$	inner wall temperature (K)
$T_{2}$	outer wall temperature (K)
$v_{z}$	axial velocity (m/s)
$v_{\theta }$	tangential velocity (m/s)
V	dimensionless velocity
z	axial coordinate (m)
*Greek symbols*	
β	degree of asymmetry
ω	rotational speed (rad/s)
Ω	temperature-difference parameter
θ	dimensionless temperature
$\theta_{m}$	dimensionless bulk mean temperature
$\theta_{mc}$	dimensionless mean temperature
ϕ	tangential coordinate
Φ	viscous-dissipation function
μ	dynamic viscosity (kg/m·s)
*Subscripts*	
f	initial fluid
m	mean
